# Effects of chlorine particle concentration on the human airway

**DOI:** 10.1007/s11051-022-05493-5

**Published:** 2022-05-20

**Authors:** Fifi N. M. Elwekeel, Xinguang Cui, Antar M. M. Abdala

**Affiliations:** 1 Faculty of Industrial Education, Helwan University, Cairo, Egypt; 2 School of Aerospace Engineering, Huazhong Science and Technology University, Wuhan, China; 3 Faculty of Engineering, Matareya Branch, Helwan University, Cairo, 11718 Egypt

**Keywords:** Environmental and health effects, Chlorine particles, Human airway, COVID-19

## Abstract

For COVID-19, chlorine has lately been utilised as a home disinfectant. Given that chlorine is hazardous to the human airway, the current research investigates the effects of chlorine mass fraction and droplet size on the human airway. The effects are investigated at chlorine mass ratios of 2% (24 ppm), 10% (120 ppm), 15% (180 ppm), and 20% (240 ppm), as well as chlorine particle diameters of 10 nm, 20 nm, 30 nm, and 50 nm, and three inhalation rates (15 l/min, 30 l/min, and 60 l/min). The results reveal that when the chlorine mass fraction is 2% and the inhalation rate is low, the chlorine volume fraction decreases. Furthermore, at 2% chlorine and a rapid breathing rate, chlorine particles are accelerated to escape into the lungs.

## Introduction

There are many different types of environmental aerosols that are inhaled and transported through the human airways. These particles may come into contact with the airway surface and cause a harmful reaction. The relation between aerosol particle sizes and bronchial illnesses has been studied extensively. Underground miners have been found to get lung cancer as a result of their exposure to ultrafine particles (less than 200 nm) (Cheng and Swift 2016). Nanoparticles in the air were more damaging and hazardous, particularly to the elderly and those with respiratory disorders (Frampton 2001; Donaldson et al. 2000; Oberdorster 2001; Kumar and Goel 2016). Underwood (Underwood 2017) and Chen et al. (Chen et al. 2017) demonstrated that there was a relation between ill health and a polluted environment.

Over the last 25 years, other research have looked into the health dangers of aerosols and the causes of early death (ICRP 1994; Cohen et al. 2017; Heal et al. 2012; Lelieveld et al. 2015). These particles have a diameter of less than 0.1 μm and contributed to air pollution. Fuel exhaust (5 to 500 nm) (Kumar and Goel 2016), smoke (140 to 500 nm) (Kittelson 1998; Bernstein 2004), and radionuclides (2 to 200 nm) (Keith 1982) are all contributors of this aerosol. The capacity of these nanoparticles to deposit in the airway was explored in these studies. Age, sex, and health status all influenced the percentage of these particles deposited (Kim and Jaques 2004; Horemans et al. 2012; Chalupa et al. 2004). For example, with a particle size of 0.04 nm, females had a higher percentage of deposition than males.

Other studies (Kan et al. 2007; Chen et al. 2012, 2011; Meng et al. 2013; Breitner et al. 2011; Li et al. 2016) looked at how much particle matter was deposited in people’s lungs in the most polluted cities. People with heart disease were more likely to die in a contaminated environment with particles ranging in size from 30 to 100 nm, according to these research.

**Fig. 1:**
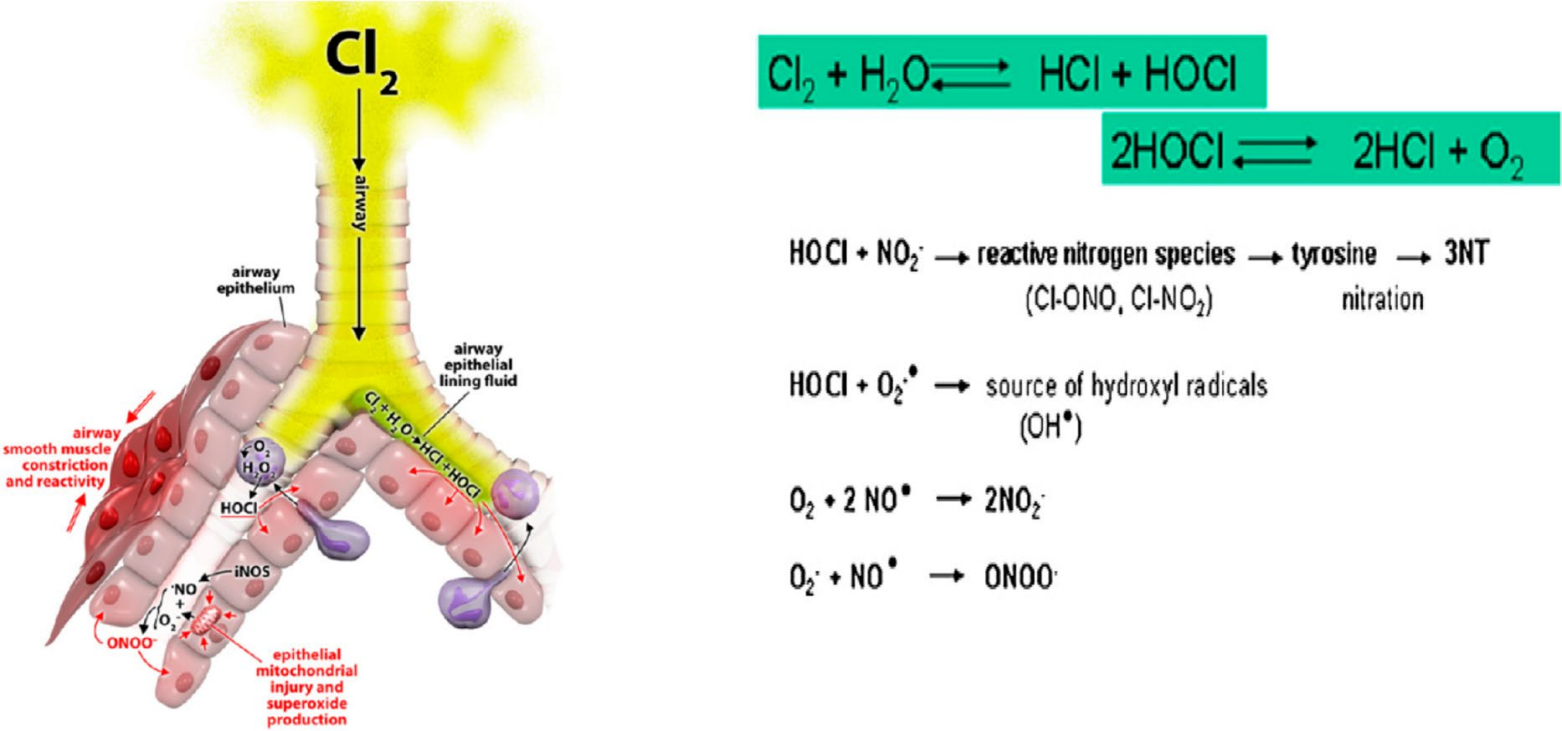
Chlorine reactions in the airway at chlorine inhalation (White and Martin 2010)

The majority of earlier investigations focused on nanoparticle deposition in the airway caused by fuel vapour and cigarette smoke. However, disinfectants are increasingly critical for coronavirus sterilisation. Chlorine is one of the most well-known disinfectants, and it is used in various concentrations, because chlorine gas is a hazardous gas that induces respiratory symptoms at 15 ppm and can be deadly at 430 ppm within 30 min (Chauhan et al. 2008). Chlorine has a variety of toxicity in the lungs, as seen in Fig. 1. When it comes into contact with mucosal surfaces and airways, it produces various acidic forms and a range of highly reactive oxidants (White and Martin 2010). As a result, the quantitative deposition of chlorine particles in the airway will be examined in this work. In addition, the effects of various chlorine concentrations and particle sizes will be described in this study.

## Methods

### Geometry

**Fig. 2:**
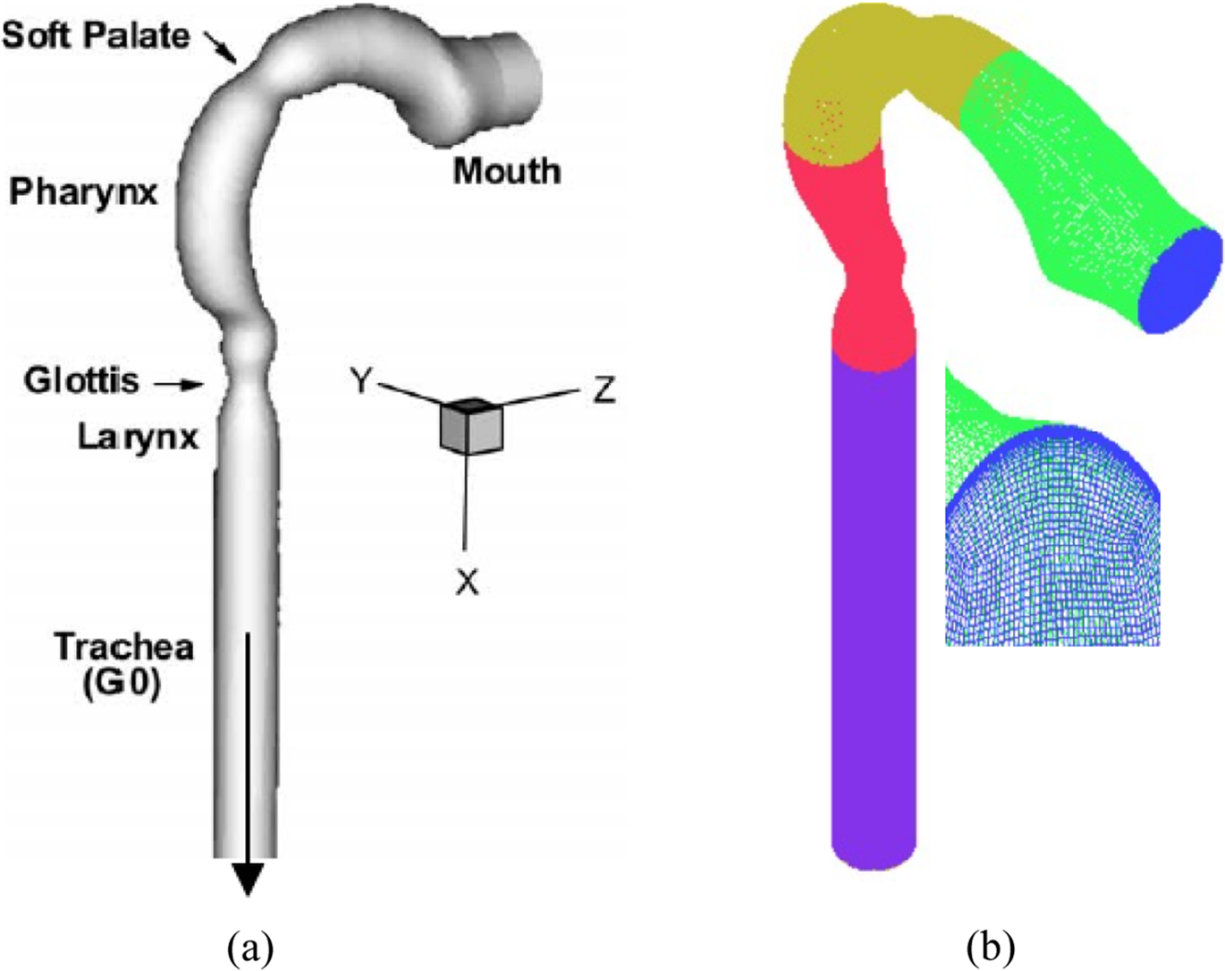
Geometry of the upper airway (Zhang and Kleinstreuer 2004): (**a**) computation domain, (**b**) mesh grid

Figure 2 depicts the oral airway configuration. The mouth cavity, pharynx, larynx, and trachea are all part of it. Cheng et al. (Cheng et al. 1997) investigated the oral airway configuration experimentally, and the 3D oral airway structural mesh is generated using the ICME Ansys software. The grid independence for airways was tested by Zhang and Kleinstreuer (Zhang and Kleinstreuer 2004) and Cui and Gutheil (Cui and Gutheil 2017, 2018); they chose 740,000 cells. The grid size will be examined at one million cells in the current study. The refine mesh is on the wall in order to capture particle deposition and keep Y^+^ below one (Fig. 2b).

### Governing equations

The governing equations will be utilised to explore the flow in the human airway, and ANSYS CFX will be used to solve these equations. The three flow rates employed in low, medium, and high breathing are 15, 30, and 60 l/min (Zhang and Kleinstreuer 2004). The continuous fluid is ideal air, while the discreated fluid is chlorine particles. The continuous fluid enters at a constant velocity (0.8, 1.6, and 3.2 m/s). Isothermal temperature (300 K), subsonic flow, 1-atm relative pressure, and low turbulence intensity characterise the flow regime. The exit pressure is zero, and the airway walls are considered to be no slip condition. The inlet velocity of the discreated fluid is set to that of the continuous fluid, and 10,000 chlorine particles (Cui and Gutheil 2017) are uniformly injected into the inlet. The mass ratio of chlorine 2%, 10%, 15%, and 20% is employed with mean particle diameters of 10 nm, 20 nm, 30 nm, and 50 nm, respectively (Zhang and Kleinstreuer 2004).

In the absence of gravity, the Euler-Euler method is used to model particle motion. The following equations are used to compute the transit of chlorine particles in breathed air.

#### Continuous fluid

Ideal air is a continuous fluid that flows into the airway with chlorine particles. This study is conducted under steady-state, incompressible, and isothermal conditions.

Continuity:

1 $$\frac{\partial {u}_{i}}{{x}_{i}}=0$$

Momentum:

2 $${u}_{j}\frac{\partial {u}_{i}}{\partial {x}_{j}}=-\frac{1}{\rho } \frac{\partial p}{\partial {x}_{i}}+v\frac{{\partial }^{2}{u}_{i}}{\partial {x}_{j}\partial {x}_{j}}$$

where *i*, *j* = 1, 2, and 3. ${u}_{i}$ is the air velocities in *x*, *y*, and *z* directions. $p, \rho , \mathrm{and} v$ are the pressure, the density, and the kinematic viscosity of air, respectively.

#### Discrete fluid (nanoparticles)

The mass transport equation of nanoparticles is (Friedlander 2000):

3 $$\frac{\partial ({u}_{j}C)}{\partial {x}_{j}}=\frac{\partial }{\partial {x}_{j}}\left(D\frac{\partial C}{\partial {x}_{j}}\right)$$

$C\mathrm{ and }D$ are the concentration and the effective diffusion coefficient of nanoparticles, respectively. The effective diffusion coefficient of nanoparticle can be calculated as (Cheng et al. 1988):

4 $$D=\frac{\left({K}_{B}T {C}_{\mathrm{slip}}\right)}{\left(3\uppi \mu {d}_{p}\right)}$$

5 $${C}_{\mathrm{slip}}=1+\frac{2{\lambda }_{m}}{{d}_{p}}\left[1.142+0.058{e}^{\left(-0.999\frac{{d}_{p}}{2{\lambda }_{m}}\right)}\right]$$

${K}_{B }, T, {C}_{\mathrm{slip}}, \mathrm{ and }{\lambda }_{m}$ are the Boltzmann constant, the air absolute temperature, the Cunningham correction, and the mean free path in the air, respectively.

The nanoparticle deposition fraction is the ratio of the mass deposited on the wall to the inlet mass flow rate, which can be calculated as (Ghalati et al. 2012):

6 $${\left({DF}_{i}\right)}_{\mathrm{nanoparticles}}=\frac{{\dot{m}}_{w,i}}{{\dot{m}}_{in}}$$

The total mass deposition ${\dot{m}}_{w,i}$ can be written as:

7 $${\dot{m}}_{w,i}=\sum_{j=1}^{N}-{\left.\rho {A}_{j }D\frac{\partial C}{\partial n}\right|}_{w,j}$$

*N* is the number of cells closest to the walls in one certain region, *n* is the normal direction to the wall, and *A*_j_ is the face area of each cell next to the walls in region *i*.

### Validation

**Fig. 3:**
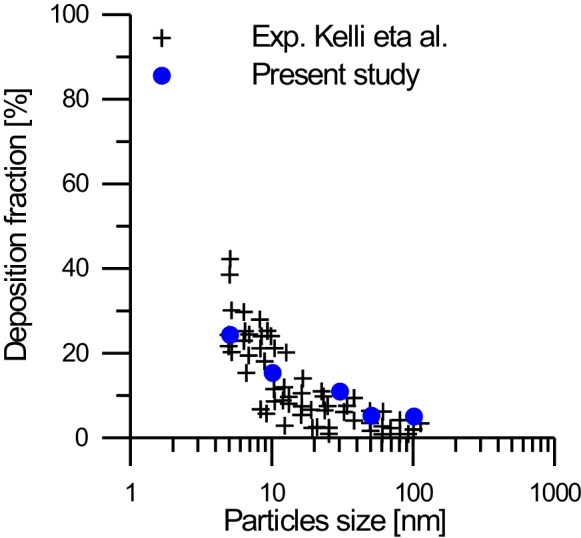
Relation between deposition fraction and particles size

To validate the numerical model with experimental results, the low Reynolds number K-ω model was adopted. Several investigations (Zhang and Kleinstreuer 2004, 2003) have identified this model as well. This model has been proved to be a viable method for capturing velocity profiles and kinetic energy disturbance of transitional flows in the upper airways’ narrow tubes. For the experimental investigation (Kelly et al. 2004) and numerical data, Fig. 3 depicts the relationship between deposition fraction and particle size. Verification is carried out at particle sizes ranging from 2 to 100 nm, with a mass fraction of 2% (24 ppm) and a flow rate of 15 l/min. The K-ω model is congruent with the experimental model in this figure; hence, it can be used in this investigation. RMS residuals of 1 * 10^−5^ are used as convergence criterion.

## Results

### Effect of respiratory flow rates at chlorine mass fraction 2%

**Fig.4:**
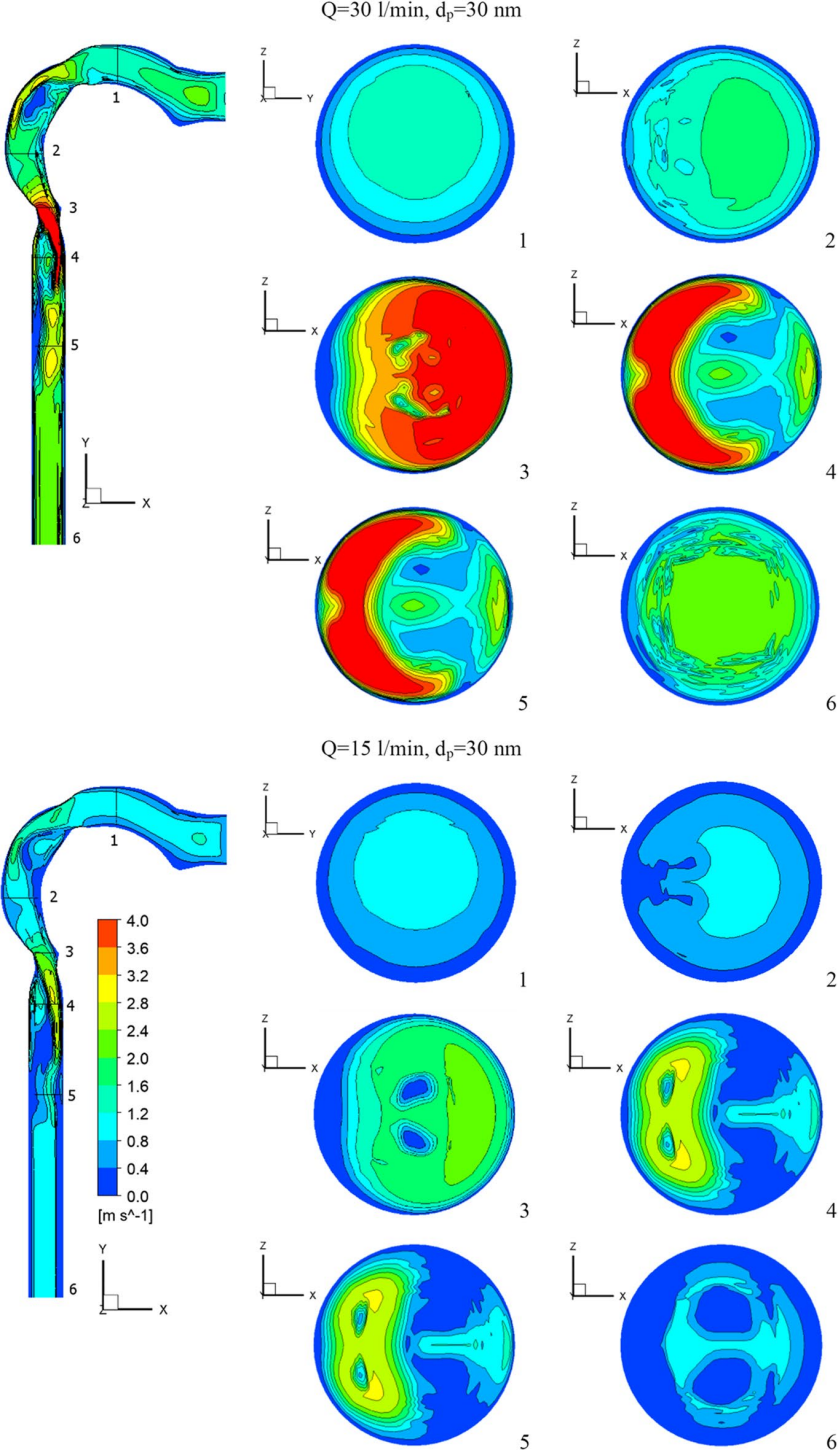

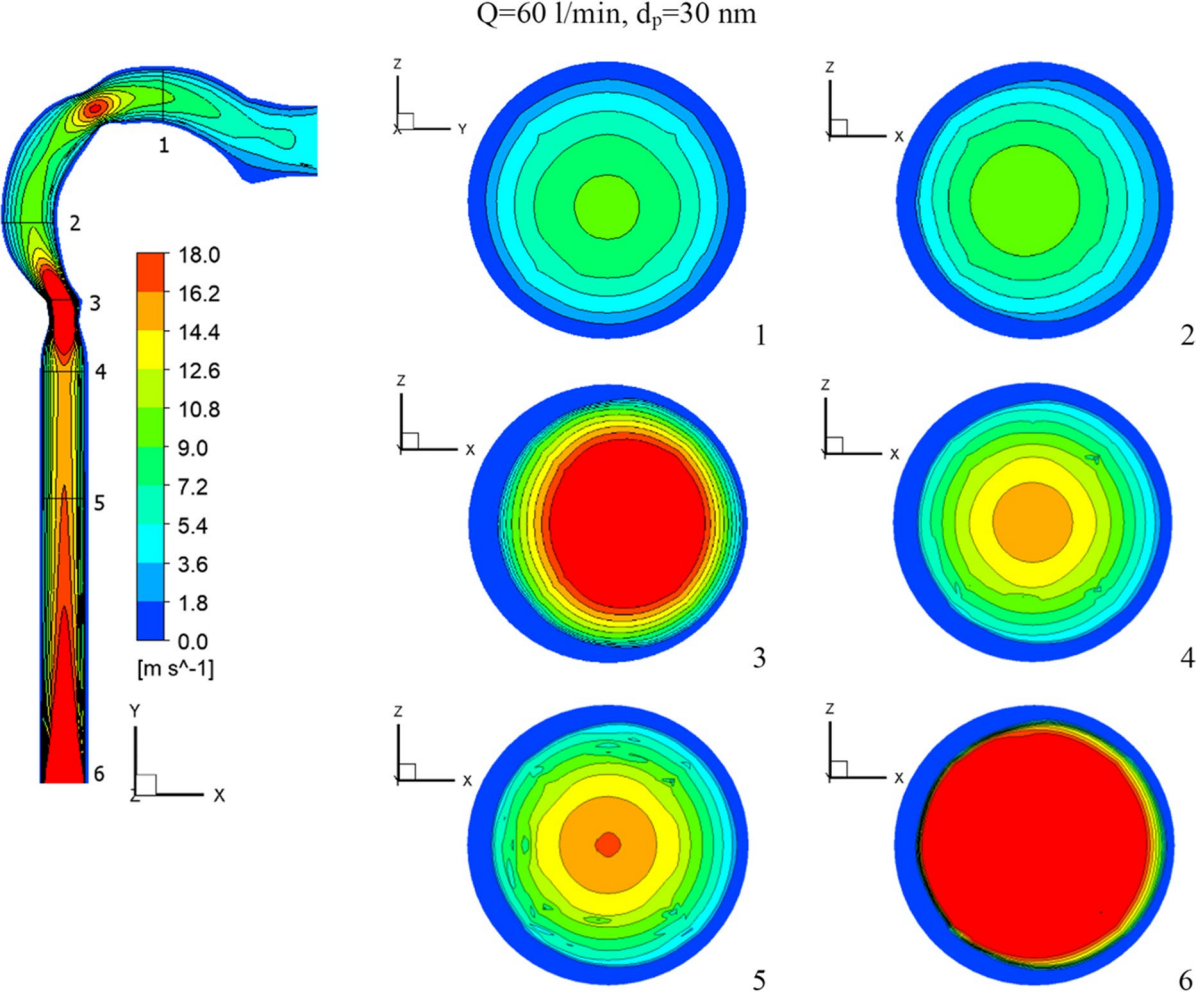
Velocity of chlorine particles in the airway

**Fig. 5:**
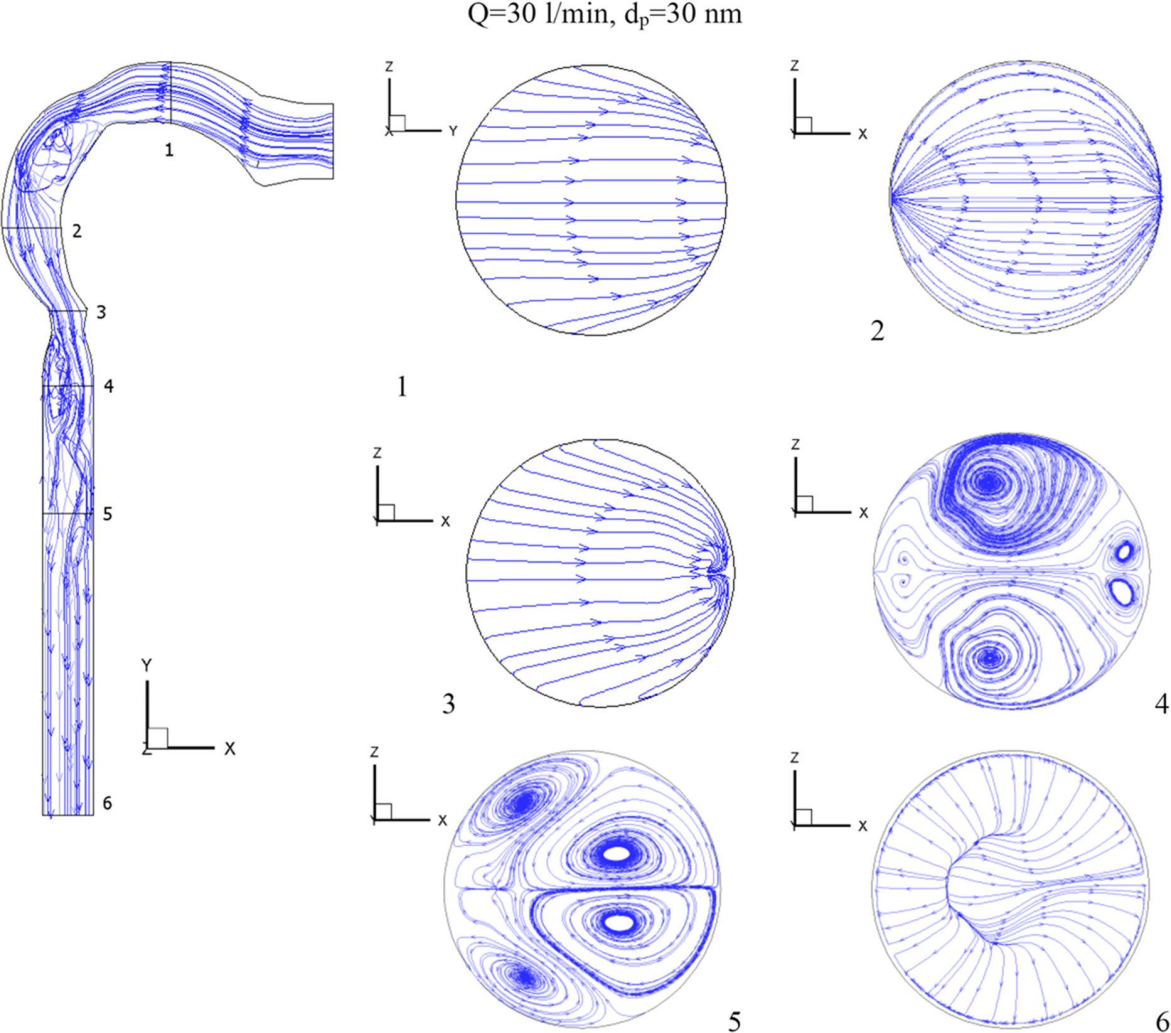

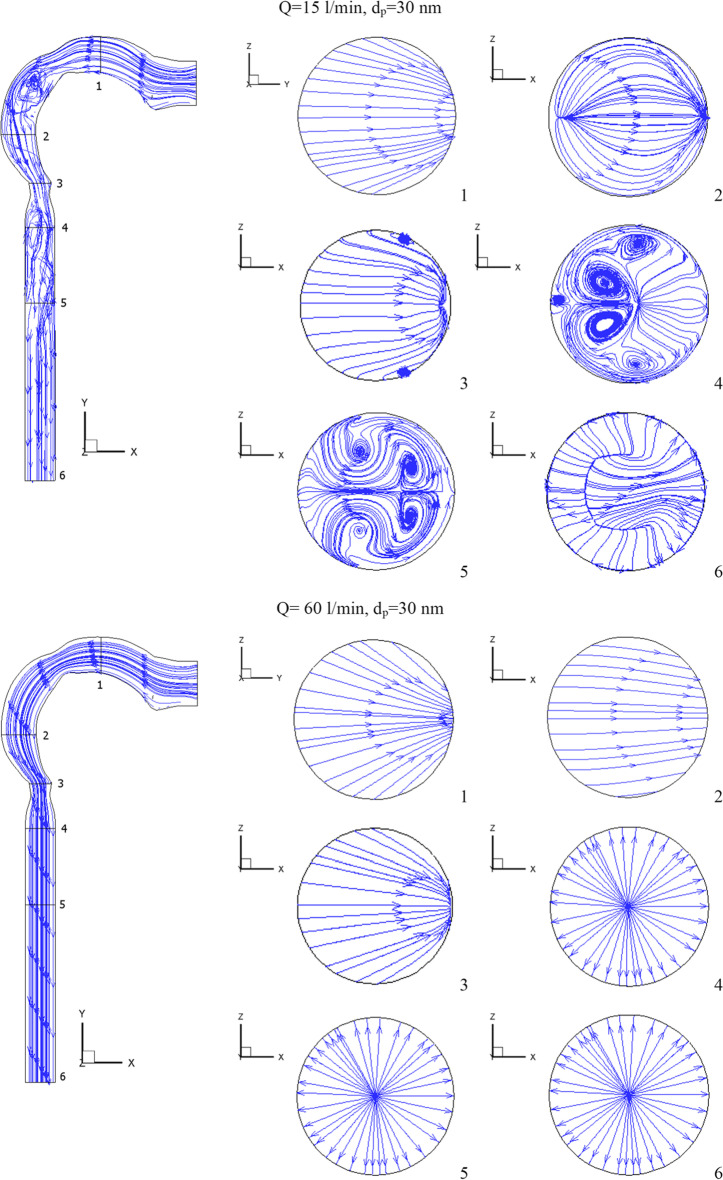
Velocity streamline in the airway

The flow of the chlorine/air mixture into the airway will be shown in this section. Inhalation rates are 15 l/min, 30 l/min, and 60 l/min. The chlorine particles have a diameter of 30 nm and a mass fraction of 2% chlorine (24 ppm). Figure 4 depicts the chlorine velocity at various locations and inhalation flow rates. With higher flow rates, the chlorine velocities increase. The velocity increases in the pharynx/larynx for low and medium inhalation rates, and the maximal location shifts due to centrifugal force (see cross-Sects. 3 and 4). The velocity is more uniform with a high inhalation rate (*Q* = 60 l/min), and the maximum velocity is in the center. As seen in Fig. 5, at inhalation flow rates of 15 and 30 l/min, the separation at the airway wall rises. As illustrated in cross-Sect. 4, changing the laryngeal cross-section causes secondary flow. The highest velocity is achieved at the center at a high inhalation flow rate of 60 l/min because the pressure gradient is driven by centrifugal force (Kleinstreuer and Zhang 2003).

**Fig. 6:**
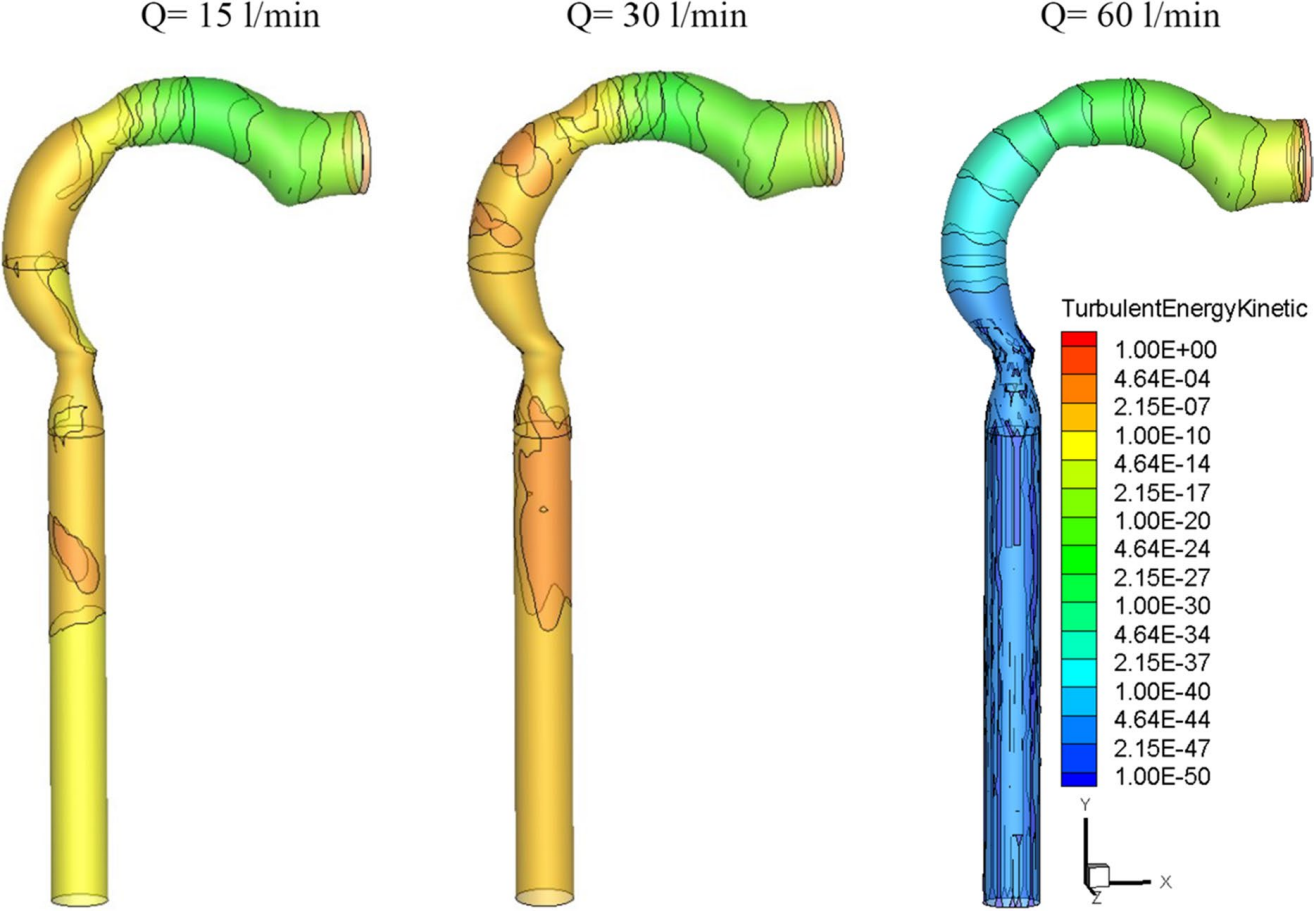
Turbulence kinetic energy with different inhalation rates and *d*_p_ = 30 nm [m^2^/s^2^]

Figures 4 and 5 further show that the Dean number has an impact on inhalation flow rates of 15 and 30 l/min. When the rate of breathing increases, the Dean number rises, causing a separation as the fluid flows into the outer wall. The greater flow rate combined with the shift in airway cross-section overcomes the adverse pressure gradient and the production of vortices at a 60 l/min inhalation rate. The turbulent kinetic energy for low, medium, and high inhalation rates is shown in Fig. 6. At *Q* = 15 l/min and *Q* = 30 l/min, turbulence strength is minimal in the oral cavity but increases in the pharynx, larynx, and trachea. The uniform velocity of a higher inhalation flow rate leads to a decrease in eddy generation and a reduction in turbulent kinetic energy.

**Fig. 7:**
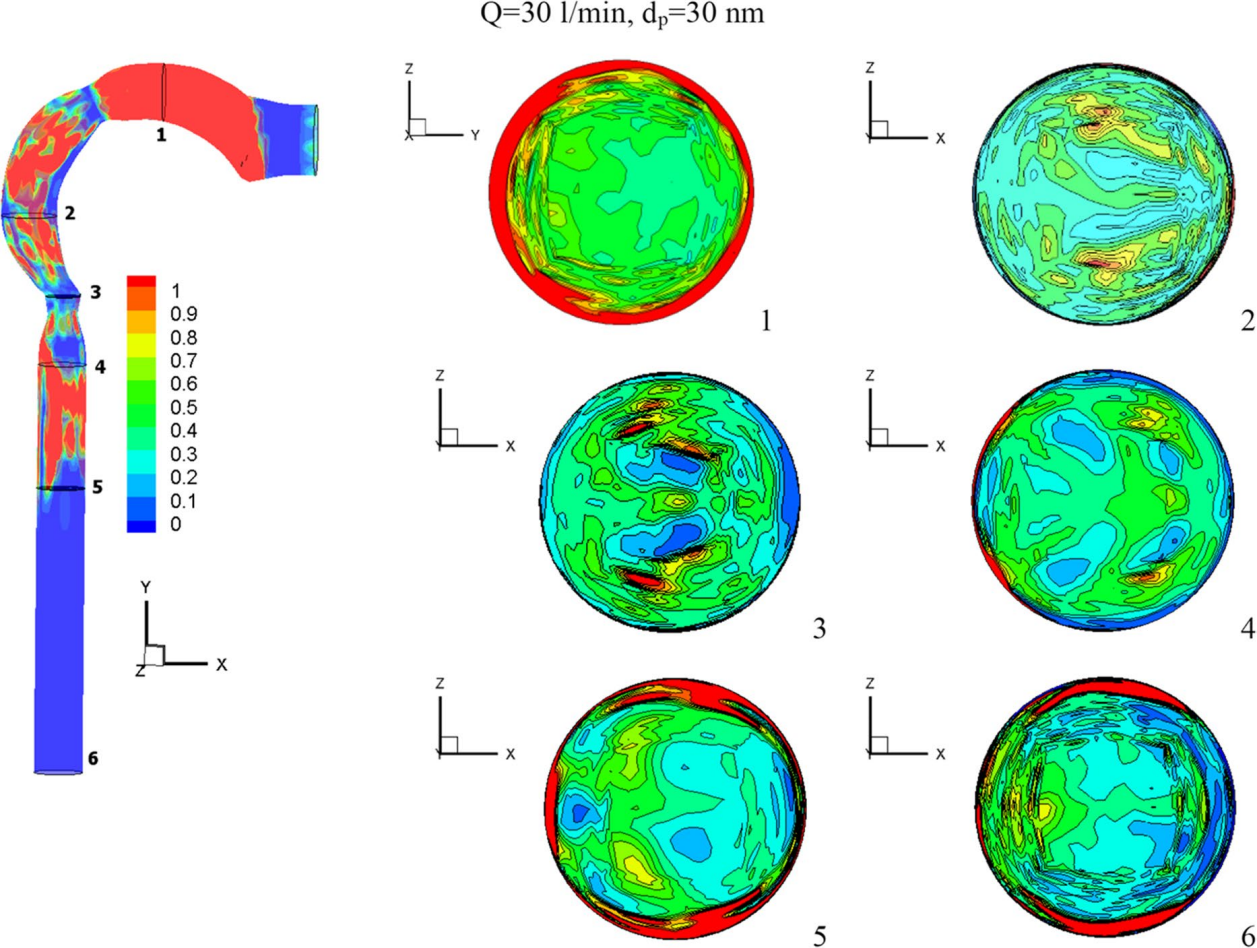

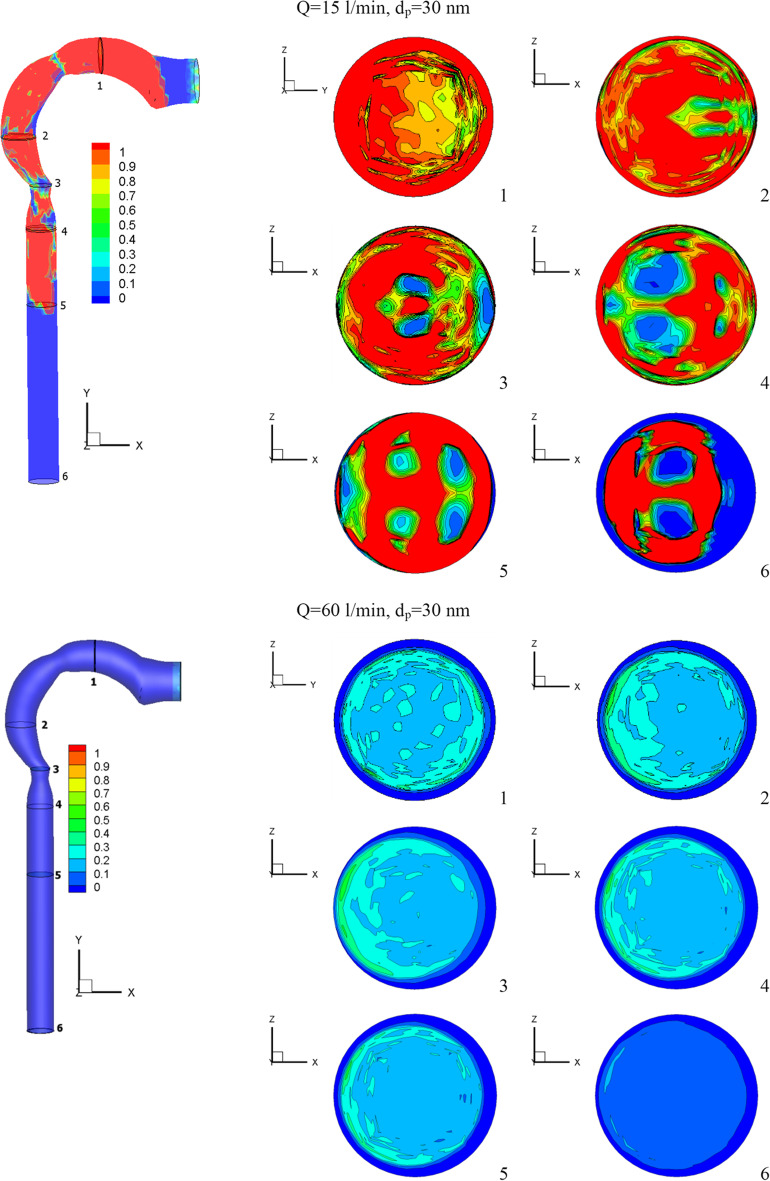
The distribution of chlorine volume fraction

Figure 7 shows the chlorine volume fraction for *Q* = 15 l/min, *Q* = 30 l/min, and *Q* = 60 l/min at particle diameter of 30 nm and mass fraction 2% (24 ppm). When the rate of inhalation rises, the volume fraction of chlorine drops. Near low inhalation (15 l/min), the chlorine concentration is spread throughout the airway, with a high value at the exit airway (cross-Sect. 6). The chlorine particles are clustered towards the wall and at their highest concentration in the mouth cavity for medium inhalation (30 l/min). The minimal velocity at the wall (see Fig. 6) results in less convection mass transfer and a lower chlorine volume percentage (Zhang and Kleinstreuer 2004).

### Effect of chlorine particle diameter

**Fig. 8:**
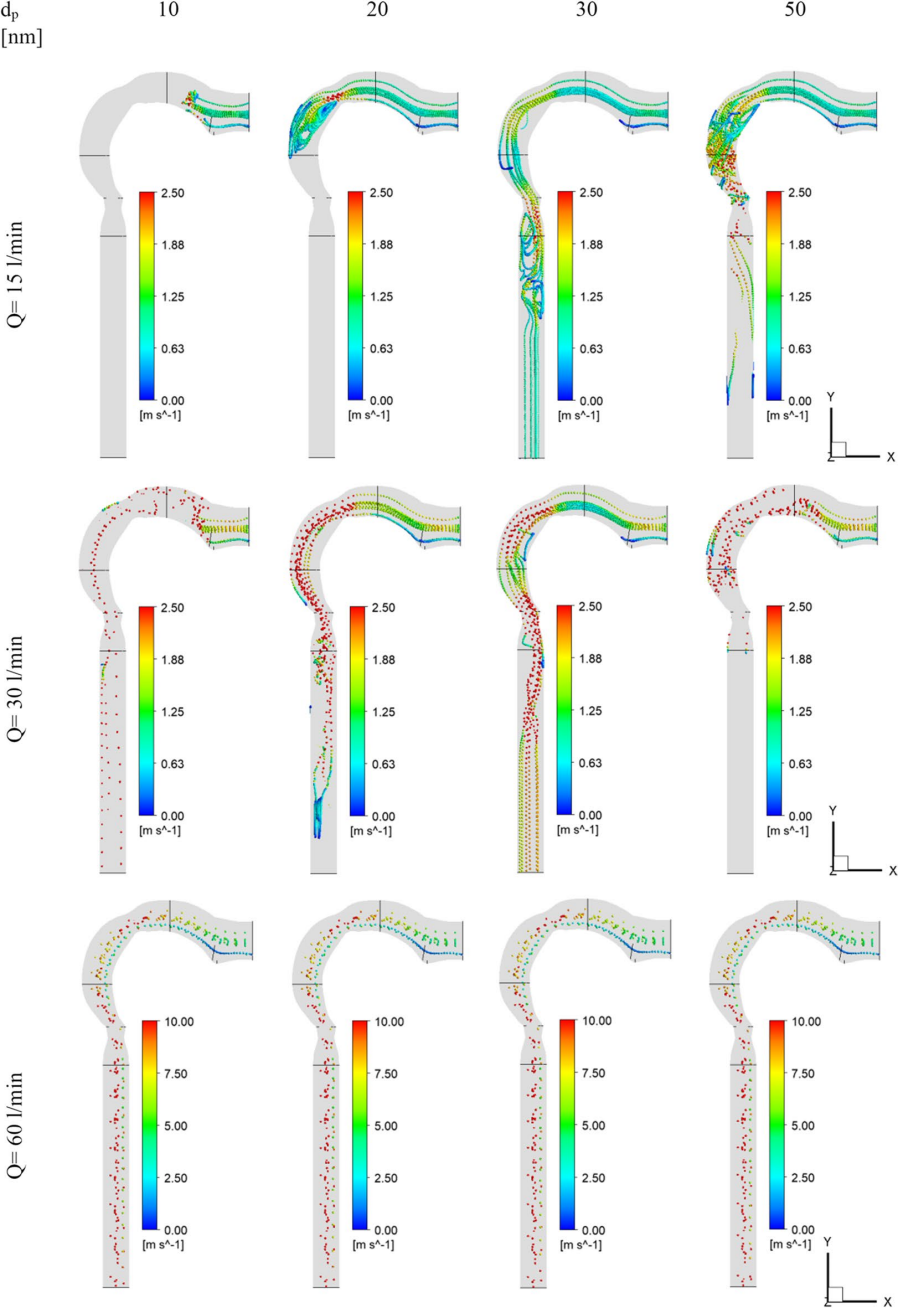
Chlorine velocity at different inhalation rates

Figure 8 depicts the average velocity of particles at various particle sizes and flow rates. The velocity of the particles drops and reaches the maximum transmission distance in the mouth cavity at a respiratory flow rate of 15 l/min, a particle size of 10 nm, and a chlorine mass fraction of 2% (24 ppm). With a lower respiratory flow rate, chlorine particles have a lower velocity, which causes more vortices in the airway. Chlorine particles are kept out of the bronchial tube by these vortices. Particles of 50 nm cause higher pharyngeal disruptions at low respiratory flow rates (15 l/min) and intermediate respiratory flow rates (30 l/min). This is due to the particles gaining a lot of kinetic energy as the cross-section of the airway changes. The particles are accelerated to escape into the bronchial tubes at a high respiratory flow rate of 60 l/min. At the pharynx and larynx inlets, the particles have a high velocity.

### Effects of chlorine mass fraction

**Fig. 9:**
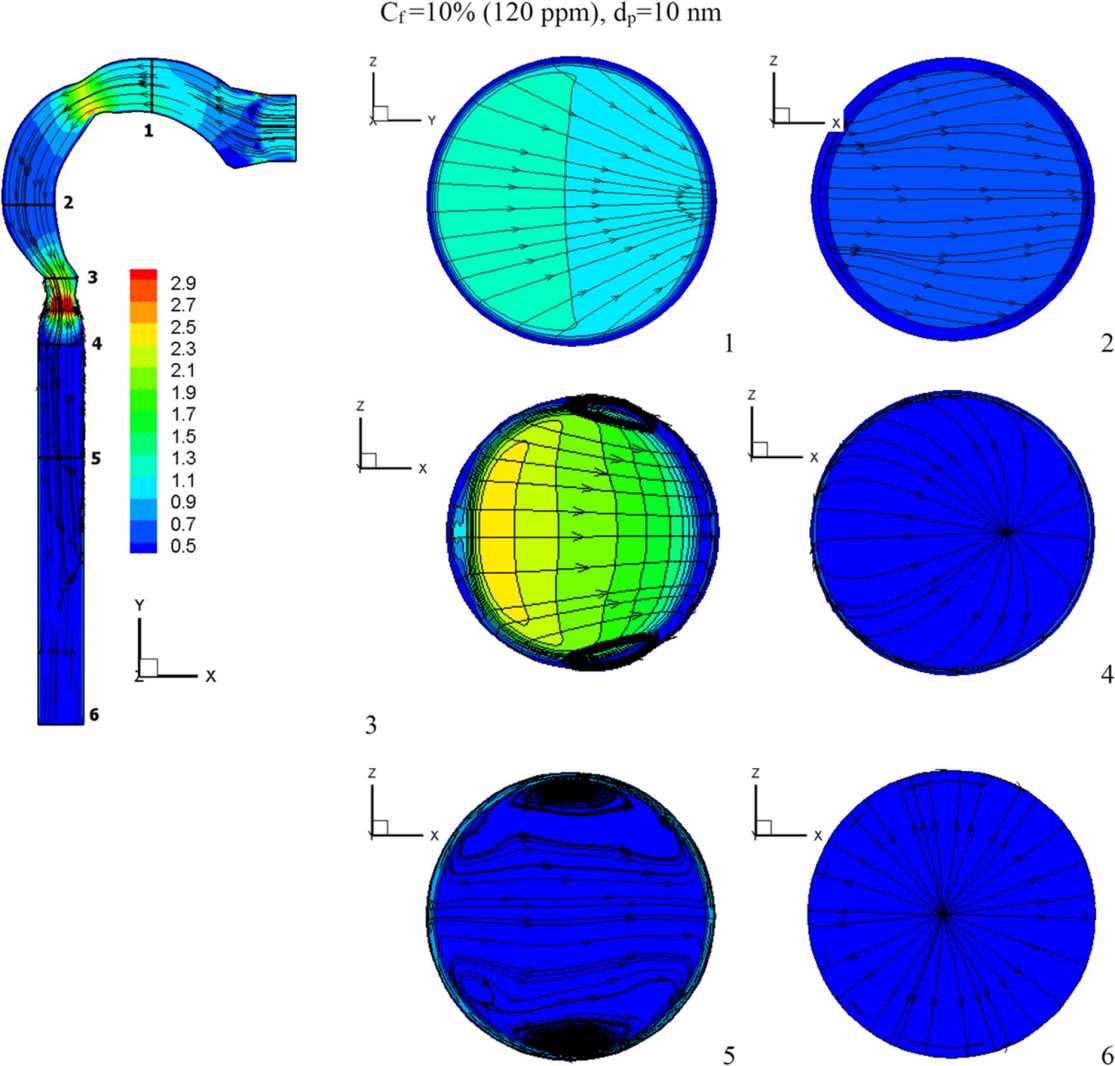

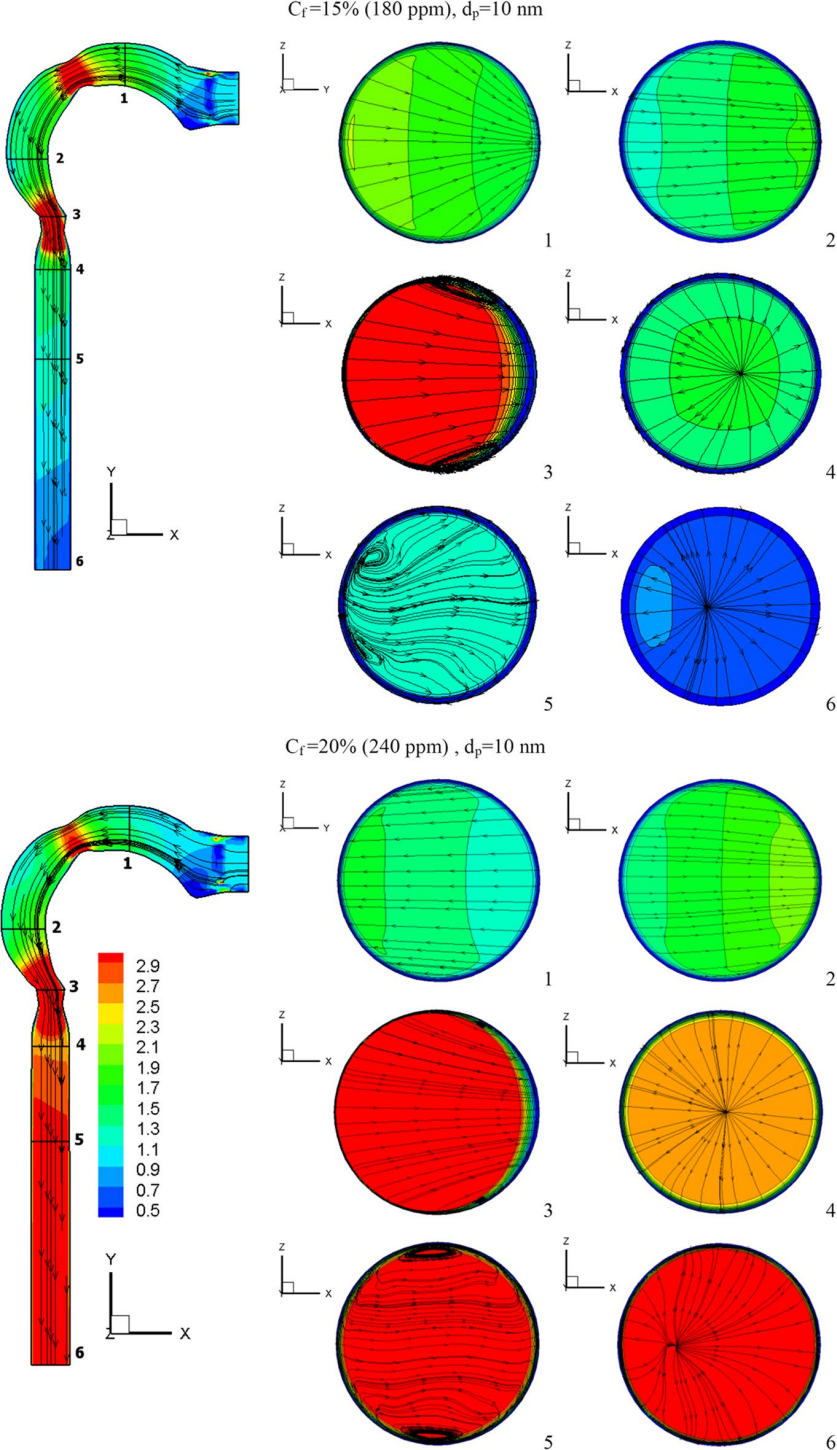
Velocity contour at varied chlorine mass fractions

**Fig. 10:**
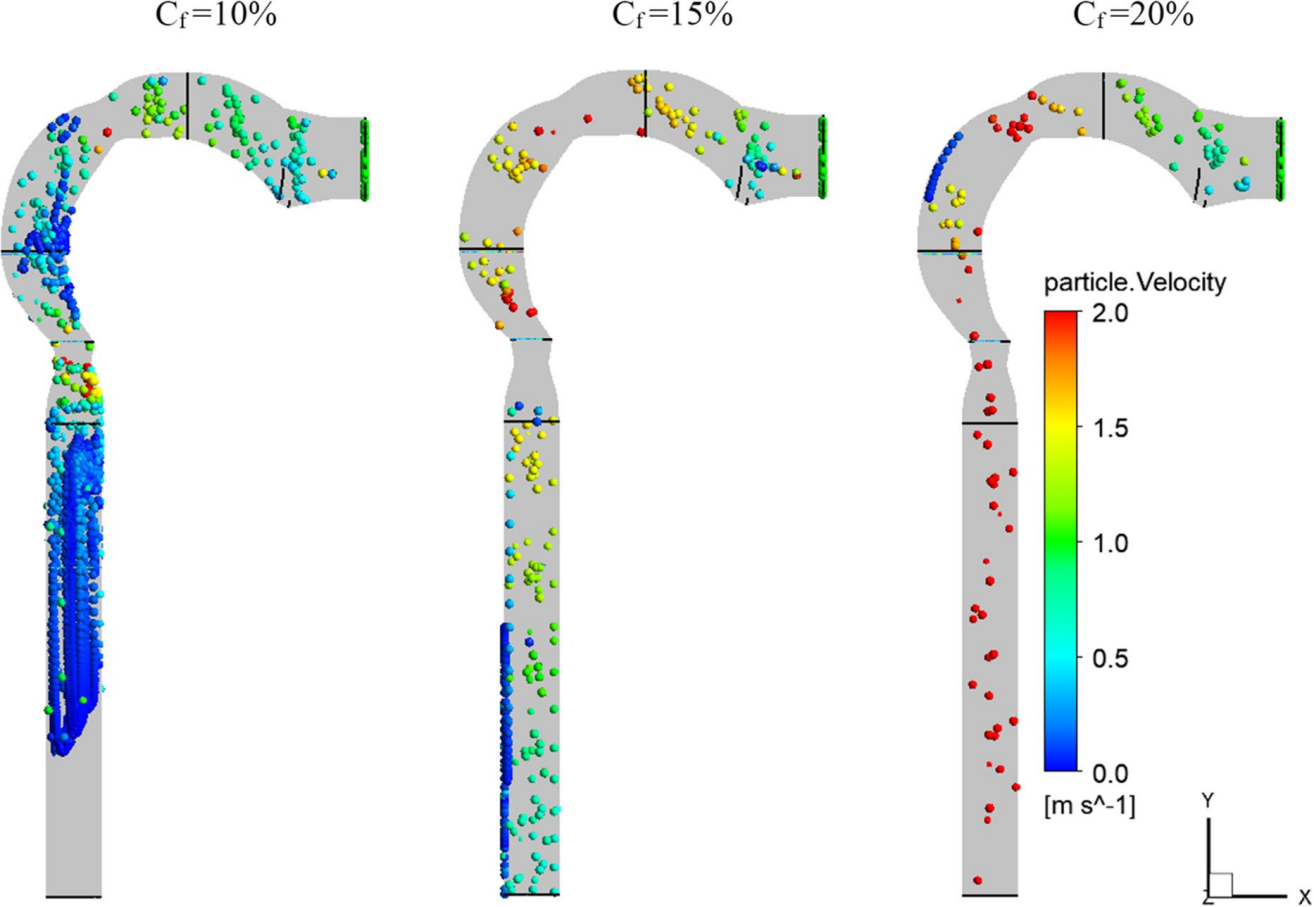
Chlorine velocity at different chlorine mass fractions and *d*_p_ = 10 nm

This section will explain how chlorine particles behave in the human airway when the mass fraction of chlorine is 10% (120 ppm), 15% (180 ppm), and 20% (240 ppm). The streamline velocity and velocity contours are shown in Fig. 9. The effects of chlorine mass fraction at a diameter of 10-nm chlorine particles and a flow rate of 15 l/min are shown in this diagram. Increased chlorine mass fraction improves velocity while reducing vortex generation. As the chlorine mass diminishes, the chlorine particles become more concentrated in the trachea, resulting in the production of additional vortices, as seen in cross-Sect. 5. As seen in Fig. 10, the high fraction of chlorine mass creates a high velocity of chlorine particles. By raising the mass fraction of chlorine, the chlorine particles accelerate the flow of liquid. The average velocities for the chlorine mass fractions of 20%, 15%, and 10% at the exit of the airway are 2.7 m/s, 0.62 m/s, and 0.31 m/s, respectively.

**Fig. 11:**
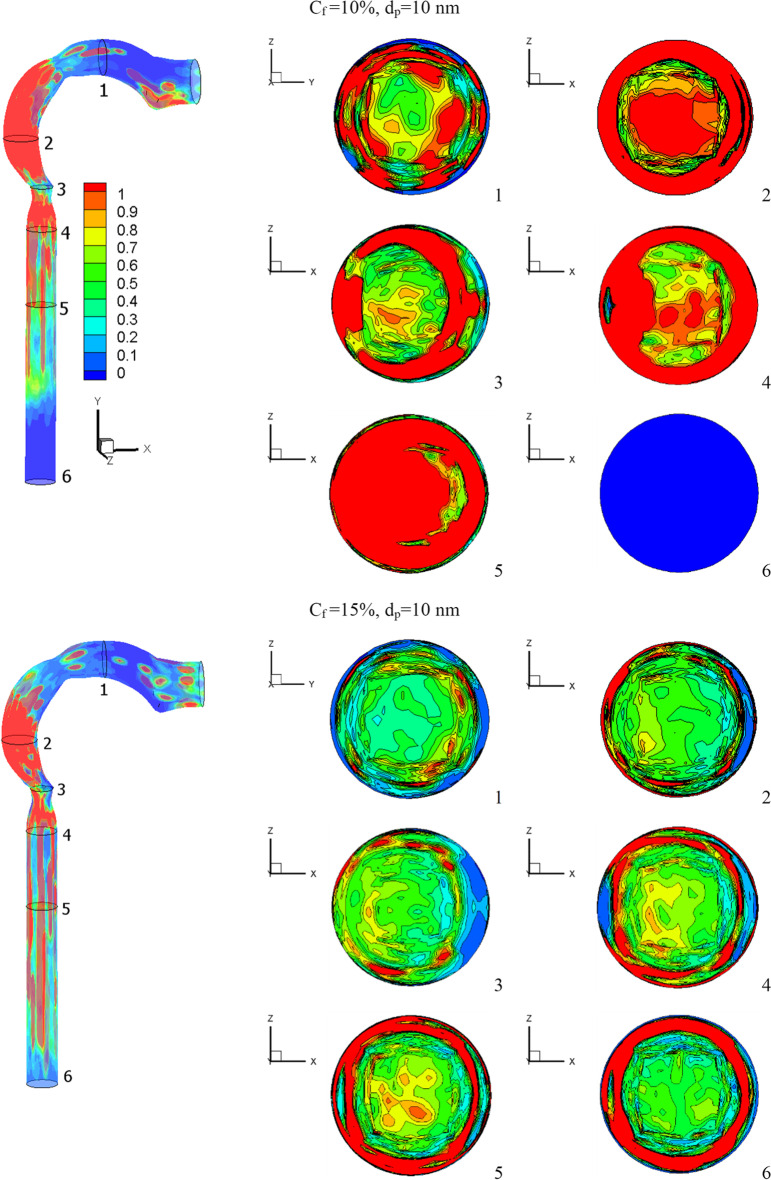

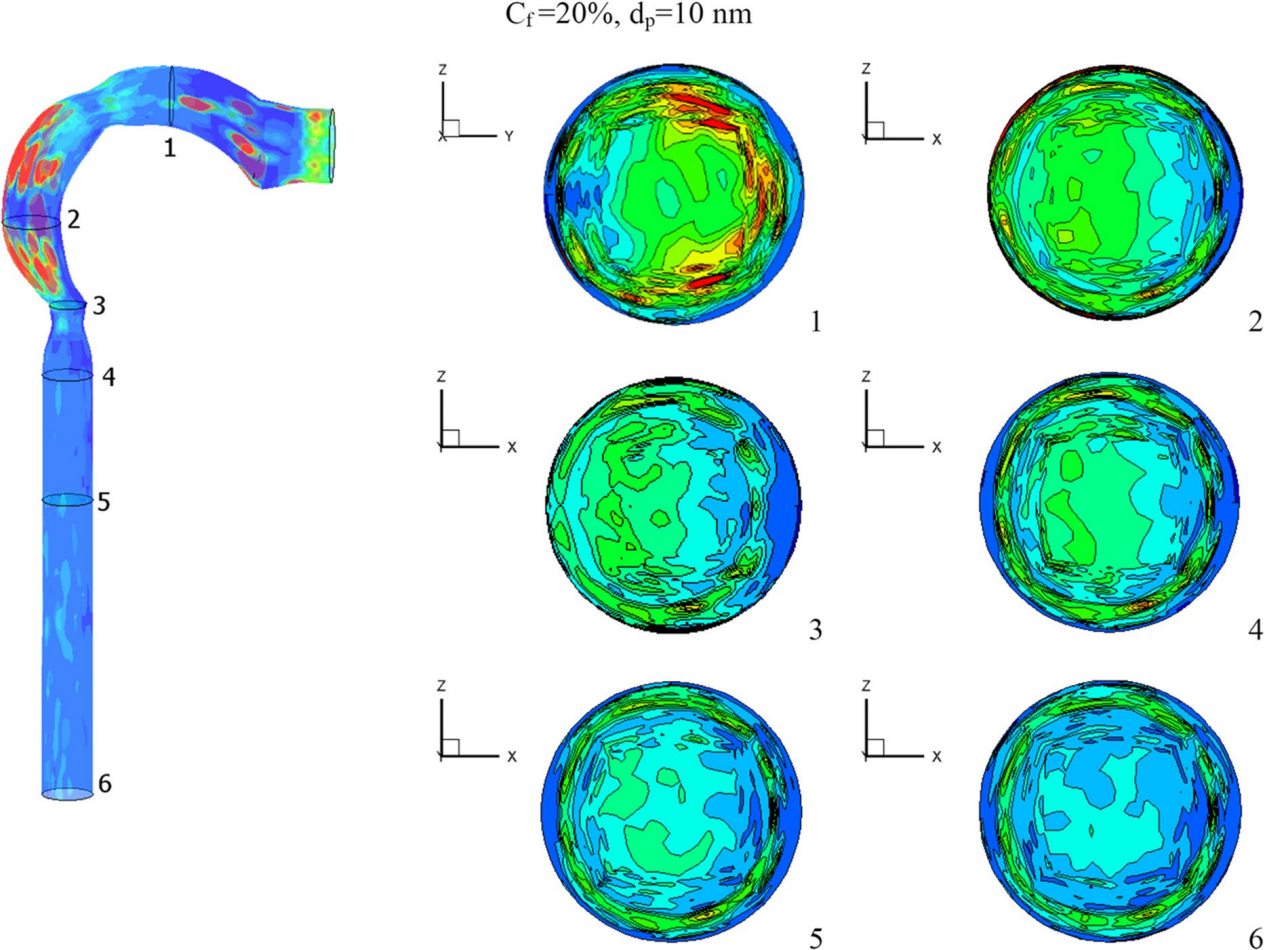
Volume fraction of chlorine at different chlorine mass fractions

The effects of chlorine mass fractions are demonstrated in Fig. 11 at a low inhalation rate of 15 l/min, with mass fractions of 10%, 15%, and 20%, and the chlorine particle diameter is 10 nm. The volumetric percentage of chlorine in the airway with varying chlorine mass fractions is shown in Fig. 11. The mass fraction of chlorine is inversely related to the volume fraction. The chlorine volume fraction concentration varies in different parts of the airway. Because chlorine particles are more stable in the airway with a mass fraction of 10% chlorine, the maximum chlorine volume is attained at cross-Sects. 1 to 5. At a 10% chlorine volume fraction, the deposition of chlorine particles is high because the particles have a decreased velocity, which aids deposition (see Fig. 10). On the other hand, chlorine particles escape into the bronchial airways at 15% and 20% chlorine concentrations.

**Fig. 12:**
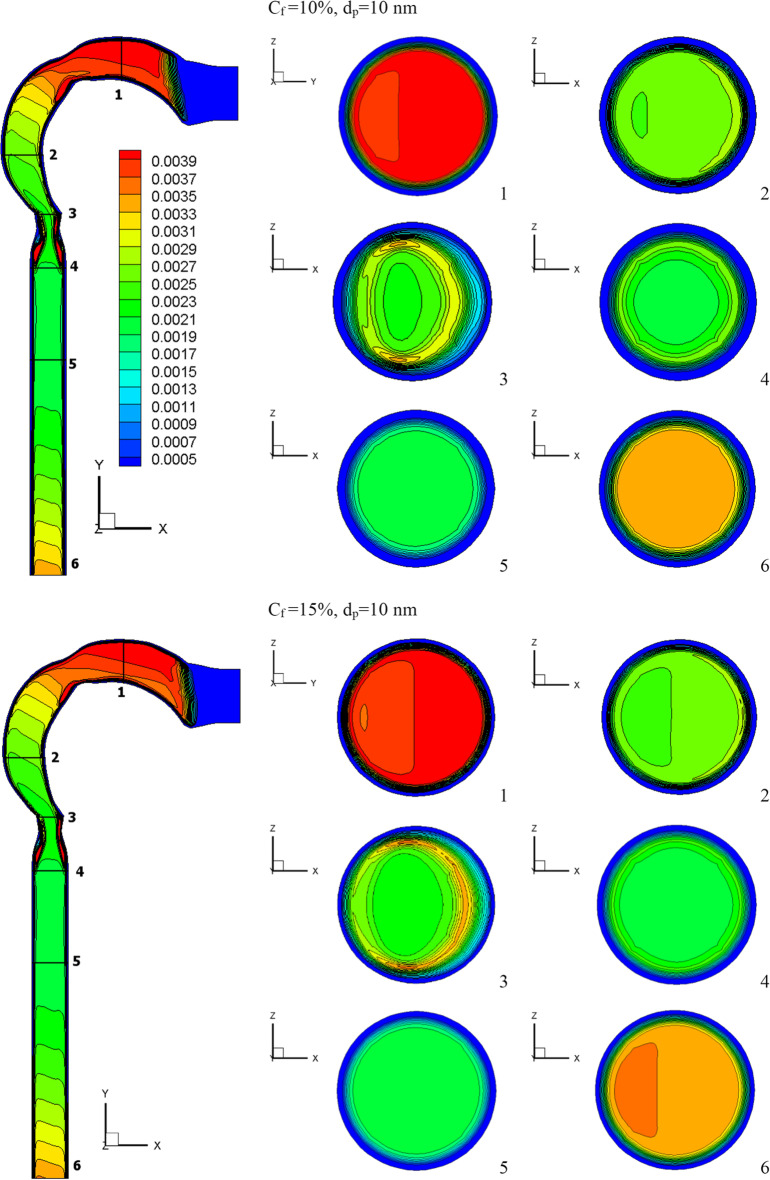

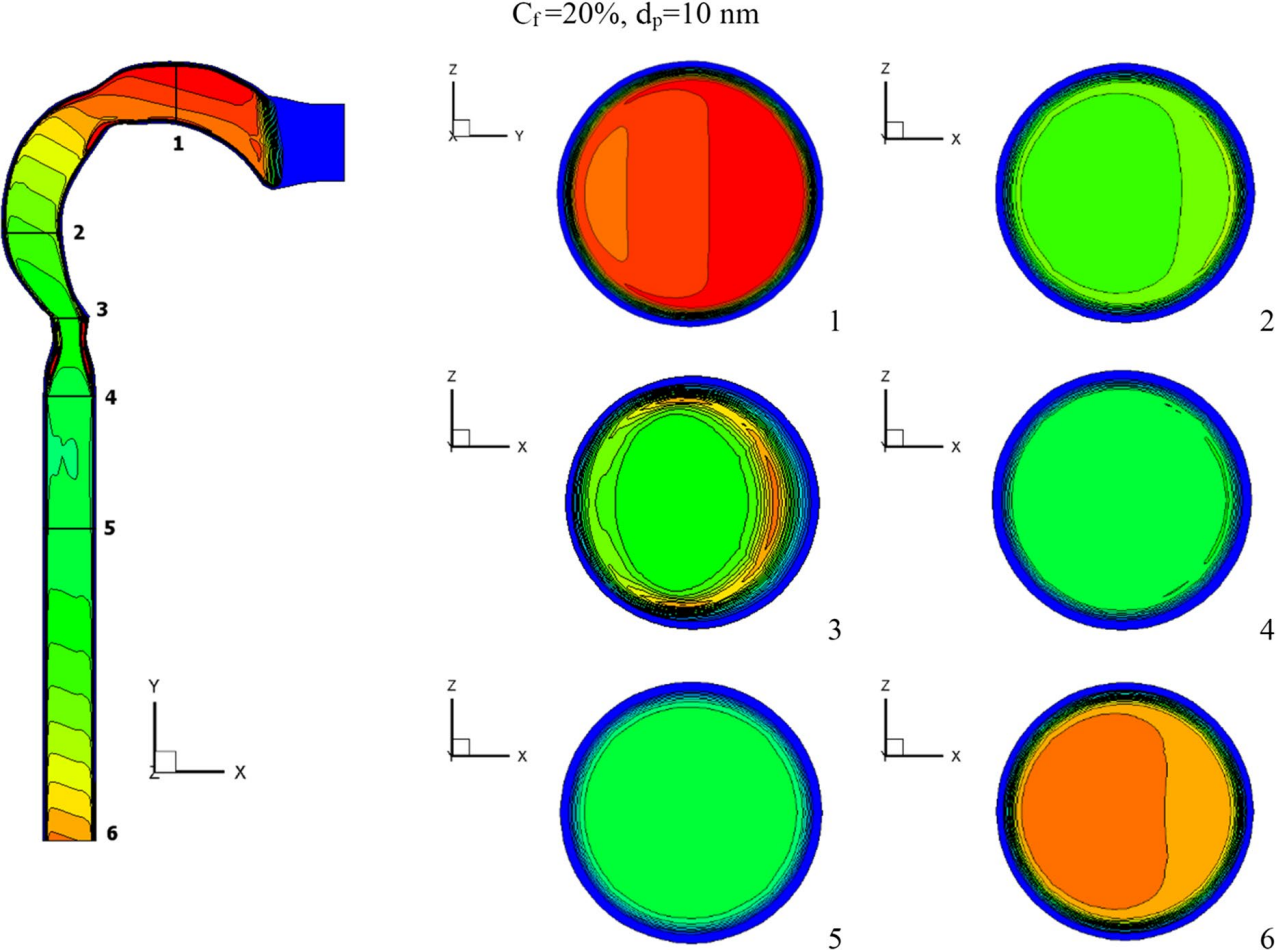
Turbulent kinetic energy at *d*_p_ = 10 nm [m^2^/s^2^]

Figure 12 depicts the impact of chlorine mass fraction on turbulent kinetic energy. The mouth cavity has a high value for turbulent kinetic energy. As can be seen, an increase in chlorine particles increases turbulent kinetic energy, particularly at the airway exits (that is matched to Fig. 10). Because chlorine particles adhere to the airway wall, the turbulent kinetic energy near the outer wall of the airway is at its lowest. The average turbulent kinetic energy at the airway exit is 14.4% and 2.2% higher at 20% chlorine than it is at 10% and 15% chlorine, respectively.

**Fig. 13:**
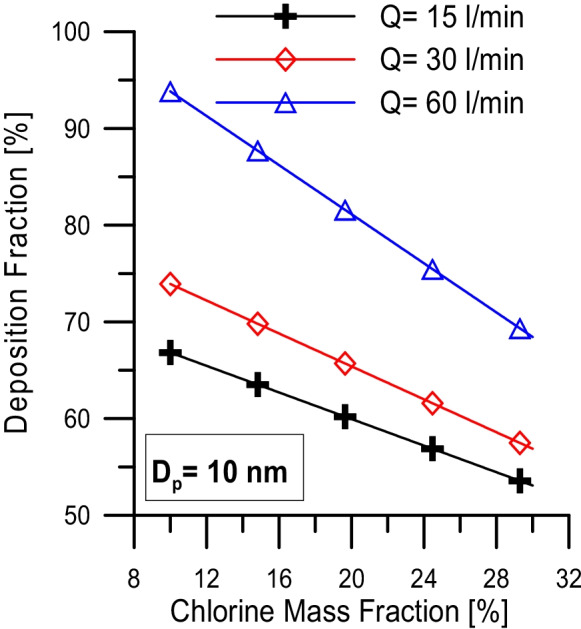
Deposition fraction of chlorine in airway with different inhalation flow rates

Figure 13 describes the relation between chlorine particle deposition efficiency and chlorine mass fraction at various inhalation flow rates. The mass fraction of chlorine has an inverse relationship with deposition efficiency, but the mass fraction of chlorine has a direct relationship with deposition efficiency. This is due to chlorine’s high density, which increases centrifugal force as the bulk of chlorine increases, speeding up the particles’ exit from the airway. In comparison to *Q* = 15 l/min, the deposition efficiency of *Q* = 60 l/min and *Q* = 30 l/min rises by around 21.8% and 4.1% at a chlorine mass fraction of 20%.

## Conclusions

The effects of chlorine particles on human airways are investigated in this study. The airway is tested using different chlorine mass fractions (2%, 10%, 15%, and 20%), different chlorine diameters (10 nm, 20 nm, 30 nm, and 50 nm), and three different inhalation rates (15 l/min, 30 l/min, and 60 l/min). The following is a summary of the research:

- The chlorine velocity rises in the pharynx/larynx at low chlorine mass fractions (2%) and inhalation rates of *Q* = 15 l/min and *Q* = 30 l/min.
- The volume fraction of chlorine reduces as the chlorine concentration (2%) falls and the breathing rate lowers.
- At a low breathing rate (*Q* = 15 l/min), lower chlorine particle diameter (10 nm), and lower chlorine mass fraction (2%), the maximum chlorine transmission distance approaches the oral cavity.
- The particles are accelerated to escape into the bronchial tubes at a high respiratory flow rate of 60 l/min, depending on the diameter of the chlorine particles (10 nm, 20 nm, 30 nm, and 50 nm) and the chlorine mass fraction (2%).
- At *Q* = 15 l/min and a diameter of 10 nm, the high chlorine mass fraction generates high velocity of chlorine particles, with average velocities of 2.7 m/s, 0.62 m/s, and 0.31 m/s, respectively, when the chlorine mass fraction is 20%, 15%, and 10%.
- As the mass fraction of chlorine increases, the turbulent kinetic energy in the outlet airway increases. The mean turbulent kinetic energy at the airway’s exit is 14.4% and 2.2% greater at 20% chlorine than at 10% and 15% chlorine, respectively.
- When compared to *Q* = 15 l/min at a chlorine mass fraction of 20%, the deposition efficiency of *Q* = 60 l/min and *Q* = 30 l/min increases by around 21.8% and 4.1%, respectively.
